# The analysis of the effect of the COVID-19 pandemic on patients with hereditary angioedema type I and type II

**DOI:** 10.1038/s41598-023-47307-1

**Published:** 2023-11-22

**Authors:** Dávid Szilágyi, Hanga Réka Horváth, Noémi Andrási, Miklós Soma Kempler, Zsuzsanna Balla, Henriette Farkas

**Affiliations:** 1 Hungarian Angioedema Center of Reference and Excellence, Department of Internal Medicine and Haematology, Semmelweis University, Budapest, Hungary; 2 Doctorate School, Semmelweis University, Budapest, Hungary; 3 Pediatric Center, Tűzoltó Street Department, Semmelweis University, Budapest, Hungary

**Keywords:** Immunological disorders, Infectious diseases, Respiratory tract diseases

## Abstract

Due to the similarity between the pathomechanism of SARS-CoV-2 infections and hereditary angioedema due to C1-inhibitor deficiency (C1-INH-HAE), a possibility emerged that C1-INH-HAE may worsen the course of the infection, or that the infection may influence the severity of angioedema (HAE) attacks in C1-INH-HAE patients. Our study aimed to evaluate the effects of the COVID-19 pandemic on the quality of life (QoL) of Hungarian C1-INH-HAE patients, and to survey the acute course of the infection, post COVID symptoms (PCS), vaccination coverage and the side effects of vaccines in this patient population. 93 patients completed our questionnaire between 1st July 2021 and 31st October 2021. In this same period and between March 2019 and March 2020, 63 patients completed the angioedema quality of life questionnaire (AE-QoL). Out of those patients infected with SARS-CoV-2 in the examined period (18/93 patients; 19%), 5% required hospitalization, 28% experienced HAE attacks in the acute phase of the infection, and 44% experienced PCS. A total number of 142 doses of vaccines were administered to the patients. Serious vaccine reactions did not occur in any case, 4 (5%) out of the 73 vaccinated patients experienced HAE attacks. No significant difference (p = 0.59) was found in the median of the AE-QoL total score, or in the number of HAE attacks prior and during the pandemic. Based on our study, HAE patients did not experience more serious SARS-CoV-2 infection, and it did not aggravate the course of HAE either. Changes in the QoL were not significant, and vaccines were safe in HAE patients.

## Introduction

Hereditary angioedema due to C1-inhibitor deficiency (C1-INH-HAE) is a rare, autosomal dominantly inherited disorder. The disease is most frequently caused by a *SERPING1* gene mutation. In type 1 HAE, the antigenic C1-INH concentration and the functional C1-INH activity decrease due to the genetic disorder, while in type 2 HAE, the concentration of C1-INH is normal, but the protein does not function properly^1,2^.

In C1-INH-HAE patients, the decreased amount or dysfunction of C1-INH periodically causes an excessive bradykinin (Bk) production^3,4^. Bk causes vasodilation and increased vascular permeability that result in recurrent edematous attacks, characteristically in the subcutaneous (head-neck, trunk, limb, gluteal region, or genitalia) and/or submucous (airway and/or gastrointestinal) tissues^1,5^. Angioedematous symptoms can vary from harmless swellings to life-threatening laryngeal edemas^6–8^.

Different factors may provoke the development of HAE attacks, like local trauma, physical exertion, menstruation, emotional stress, ACE-inhibitors, taking oral estrogen-containing contraceptives, weather change and infectious diseases^9^.

In C1-INH-HAE therapy we differentiate acute therapy, short-term prophylaxis (STP), and long-term prophylaxis (LTP). The most frequently used acute treatments are the C1-INH concentrates derived from human plasma (pdC1-INH), or C1-INH made by recombinant technology; these exert their effect by supplementing the missing C1-INH protein. Other agents, like the B2R antagonist icatibant or kallikrein inhibitor ecallantide, exist^10^. The aim of STP is most often to prevent the attacks occurring due to smaller medical interventions and operations by injecting pdC1-INH^11^. The purpose of LTP is to decrease the frequency of HAE attacks. The following can be given as first line treatments: pdC1-INH, plasma kallikrein inhibitors: lanadelumab, berotralstat. As second-line treatment, danazol or tranexamic acid can be used^11,12^.

SARS-CoV-2 is a positive-strand RNA virus responsible for an airway infection that can show the clinical picture from mild flu-like symptoms to ARDS or even, in some cases, death^13–15^. The virus, spread from animals to humans, was first discovered in December 2019 in Wuhan province of China, and from there, with increasing infection numbers, it became a pandemic in 2020^16^. In Hungary, the first cases were reported in March 2020^17^.

By post COVID symptoms (PCS), the WHO defines the symptom or syndrome that occurs 3 months from the onset of the first clinical symptom or diagnosis of SARS-CoV-2 infection; lasts for at least 2 months and has an impact on everyday functioning. This symptom may be new following initial recovery from an acute infection or persist from the acute illness. Symptoms may include tiredness, shortness of breath, chest discomfort, cough, loss of smell, dysgeusia, joint pain, loss of appetite, dizziness, muscle pain, insomnia, gastrointestinal symptoms, posttraumatic stress disorder, depression, memory problems, concentration problems, anxiety, headache. The symptoms may fluctuate, and relapses may also occur^18–20^.

To stop the COVID-19 pandemic and to reduce more severe course infections, in the beginning of 2021, the vaccines against SARS-CoV-2 occurred. In Hungary, the population could choose from six types of vaccines: mRNA vaccines Comirnaty and Moderna COVID-19; adenovirus vector-based Gam-COVID-Vac, Vaxzevria, and Janssen vaccines (the latter occurred later); and the Sinopharm vaccine containing the inactivated virus. The reactions few days after the administration of the vaccines were similar in case of all vaccines. The most common side effects included local reactions, tiredness, headache, bad mood, joint pain, shivering, hyperthermia or fever, gastrointestinal symptoms, airway symptoms, nervous system symptoms and chest pain^21,22^.

Multiple connections were found between C1-INH-HAE and SARS-CoV-2 infection. First, in July 2020 Xu et al. suggested the possibility that the course of SARS-CoV-2 may be more severe in C1-INH-HAE patients, and that the infection may aggravate HAE symptoms, or it may provoke angioedematous attacks in symptomless C1-INH-HAE patients^23^. Also, in July 2020, Garvin et al. were the first to describe the pathomechanism of the Bk storm in SARS-CoV-2 infection, as a possible etiology behind the more severe infections; this further strengthened the possible connection between the two illnesses. Besides ACE2 decrease, the smaller decrease in ACE levels may also play a role in the increase of Bk during the Bk storm, since Bk is the substrate of ACE, which may accumulate by avoiding breakdown^24^.

The COVID-19 pandemic brought other difficulties to the everyday life of people besides the SARS-CoV-2 disease, like quarantine, or the possible change of the workplace, work site, or income. These are social changes that can substantially change the everyday life and habits of people and may result in less or more stress^25–27^.

The aim of our study was to survey the course of SARS-CoV-2 infection in C1-INH-HAE patients, and to analyze the angioedematous attacks during the acute phase of the infection and the potentially occurring PCS. Our other objective was to analyze the vaccination against SARS-CoV-2, the change in the QoL of patients and the availability of HAE medications and treating physicians.

## Methods

### Patient population

Patients over 18 years with type 1 or 2 C1-INH-HAE were enrolled, who are treated in the Hungarian Angioedema Center of Reference and Excellence of Semmelweis University, Department of Internal Medicine and Hematology.

### Examined period

The examined pandemic period was from 1st September 2020 to 30th April 2021, which covered the second and third wave of COVID-19 in Hungary. In these eight months, case numbers significantly increased, and restrictions were introduced. The restrictions included entry restrictions, mandatory mask wearing in closed areas and public transportation; visiting ban in hospitals and nursing homes; food services and nightclubs were to be closed by 11 PM, and it was prohibited to conduct an event with more than 500 attendees.

### Questionnaires used for the study

We used two questionnaires in our study. The first was assembled according to our objectives by our work group, while the second was the Hungarian AE-QoL (Angioedema Quality of Life Questionnaire) used for the evaluation of the QoL.

The former questionnaire asked about the course of SARS-CoV-2 infection, the duration and the required treatment of certain symptoms, in order to establish the effects of HAE on the infection. Moreover, we analyzed the incidence and course of angioedematous attacks occurring during the acute phase of the infection. PCS were recorded according to the criteria specified by the WHO^15^. We investigated if there are PCS characteristic of HAE patients, how frequent their occurrence is and how long certain symptoms persist. We analyzed the vaccination with COVID-19 vaccines and investigated the acute reactions occurring in 48 h after the administration of the vaccine. We gave special attention to the angioedematous attacks occurring after the vaccination. Our patients keep an HAE diary, where they document the number and quality of HAE attacks. We also compared these data with the questionnaire in order to minimize memory bias. Furthermore, we asked about the change in the availability of HAE treating physicians and medications. The questionnaire was completed via telephone, between 20 June 2021 and 01 October 2021.

The AE-QoL is an internationally used and validated questionnaire that surveys the QoL of HAE patients. It analyzes the four weeks prior to completion. The test contains 17 questions which analyze QoL in 4 domains: Function, Fatigue/Mood, Fear/Shame, Food. Each question can be given 5 kinds of answers, to which points are assigned (never—1; rarely—2; occasionally—3; often—4; very often—5). The domains contain different number of questions: Function has 4; Fatigue/Mood has 5; Fear/Shame has 6 and Food contains 2. The partial score of certain domains, and the total score of the test was calculated with the (received points − number of questions)/(the maximum receivable points − the minimal receivable points) × 100 formula. The achieved score can therefore be between 0 and 100. The higher the achieved score, the worse, the lower, the better the measured QoL^27^. The questionnaires were completed in person, both prior to and during the pandemic, specifically examining the period between 1st September 2020 and 30th April 2021. For those who were infected with SARS-CoV-2, the questionnaire examined the course of their infection. The minimal clinically important difference (MCID) in the AE-QoL was 6 points^28^.

### Statistical analysis

To compare the AE-QoL scores, we used GraphPad Prism 5.0 (GraphPad Software, San Diego, CA, USA). The comparison of the AE-QoL total score and domains was performed with Mann–Whitney test, while the relation between the AE-QoL results and the number of attacks was done with Spearman correlation. A significance level of p < 0.05 was used in all statistical tests.

### Ethics approval and consent to participate

The study protocol was approved by the Regional, Institutional Scientific and Research Ethics Committee of Semmelweis University (ethics approval number 19068/2018/EKU), and we acted in accordance with the Declaration of Helsinki and obtained informed consent from the participants.

## Results

Out of the 93 patients (M/F: 35/58; min–max age: 18–79 years, median age: 48 years), 18 patients were infected with SARS-CoV-2 (M/F: 8/10, min–max age: 22–73 years; median age: 44.5 years) during the examined period (01 September 2020–30th April 2021). LTP was given to 22 patients (21 received danazol, 1 received iv. pd C1-INH concentrate).

Out of 93 patients, 61 completed the AE-QoL questionnaire both in the beginning of the Hungarian epidemic (March 2020) and during the examined period.

### Survey of the SARS-CoV-2 infected patients

**Figure 1:**
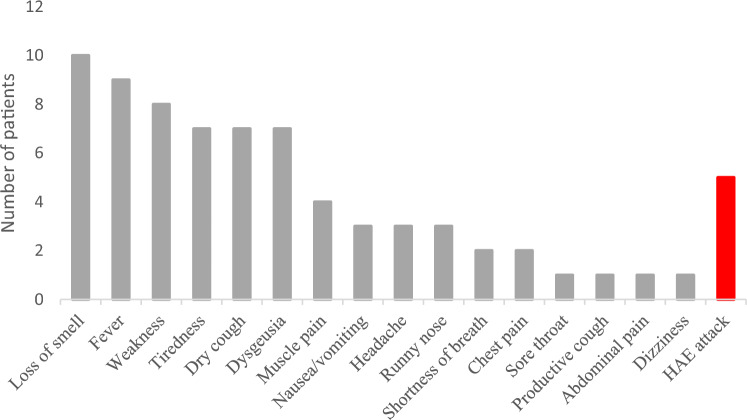
Frequency of SARS-CoV-2 infection symptoms. *The* most common symptom of the SARS-CoV-2 infection was the loss of smell, which was present in 10 patients (55.5%)*,* followed by fever (9 patients*;* 50%), and weakness (8 patients*;* 44.4%). 5 patients (27.8%) experienced at least one HAE attack during the acute phase of the infection. The localization of the attack was either abdominal or in the extremities*.*

Out of the 18 patients who became infected with SARS-CoV-2, 15 had symptoms, and the duration of the symptomatic period was 8 days on average. In 6 patients, the diagnosis was established by a positive polymerase chain reaction (PCR) test, 6 patients were the contacts of infected people diagnosed with positive tests; two patients had positive antigen rapid tests, while in 4 cases the infection was verified with an ex-post positive serological test. The symptoms are shown on Fig. 1. Out of those who were infected, only 1 (5.6%), a 65-year-old male patient needed hospitalization. The received results are influenced by the fact that 12 of the 18 patients (67%) did not have any comorbidities besides C1-INH-HAE, 4 patients (22%) were treated with hypertension, including the patient who was hospitalized, 1 (5.6%) patient was treated with hyperthyroidism and 1 (5.6%) with anxiety disorders. The median age of the infected patients is also in favor of the milder disease course. Table 1 presents the way of verifying the infection, the duration of symptoms; the number and location of HAE attacks during the infection; potential co-morbidities; the used therapy for the infection and the therapy for the HAE attacks in SARS-CoV-2 infected patients.

**Table 1:** Presentation of the clinical data of hereditary angioedema patients who underwent SARS-CoV-2 infection.

Patient Age, sex	Verification of the infection	Symptoms (duration in days)	HAE attacks during the infection	Comorbidity, therapy of infection	HAE therapy
1. 36/F	Close contact	Dry cough (2), tiredness (7)	3 abdominal	None, did not require treatment	pdC1-INH as needed
2. 22/F	Serology	Asymptomatic	Never had an attack	None	pdC1-INH as needed
3. 36/F	Close contact	Fever (7), dry cough (7), shortness of breath (7), tiredness (8), weakness (7), loss of smell and dysgeusia (21)	No attack during the infection	None, did not require treatment	pdC1-INH as needed, icatibant
4. 46/M	PCR test	Dry cough (3), shortness of breath (1), dysgeusia (1), weakness (5)	Never had an attack	Hypertension, did not require treatment	pdC1-INH as needed
5. 39/M	Close contact	Fever (1), dry cough (1), weakness(1), loss of smell(1)	1 extremity	None, did not require treatment	danazol LTP, icatibant as needed, rC1-INH
6. 44/F	Serology	Loss of smell and dysgeusia*	No attack for 2 years	None, did not require treatment	pdC1-INH as needed
7. 67/M	Serology	Asymptomatic	No attack during the infection	None, did not require treatment	pdC1-INH as needed
8. 73/M	PCR test	Fever (7)	Never had an attack	None, antibiotic treatment	pdC1-INH as needed
9. 65/M	PCR test	Fever (17), throat pain (7), gastrointestinal symptoms (7)	No attack for 15 years	Hypertension, hospitalization, oxygen therapy, antibiotic treatment	pdC1-INH as needed
10. 51/F	PCR test	Fever (6), tiredness (6), weakness (6), loss of smell (21)	3 Abdominal 1 Extremity 1 Genitalia	None, favipiravir treatment	pdC1-INH as needed, icatibant
11. 43/F	Serology	Loss of smell and dysgeusia (60)	No attack for 8 years	None, did not require treatment	pdC1-INH as needed
12. 42/M	Close contact	Fever(1), dry cough (2), runny nose (2)	No attack for 2 years	None, did not require treatment	danazol LTP
13. 72/F	Ag rapid test	Dizziness (3)	No attack during the infection	Anxiety disorder, no treatment needed	danazol LTP, icatibant as needed
14. 58/M	Close contact	Dry cough (3), tiredness (7), muscle pain (7), weakness (7), loss of smell and dysgeusia (7), abdominal pain (7)	Never had an attack	Hypertension, did not require treatment	pdC1-INH as needed
15. 33/F	Ag rapid test	Loss of smell*	1 Extremity 1 Abdominal	None, did not require treatment	pdC1-INH as needed
16. 55/F	PCR test	Fever (1), dry cough (7), tiredness (7), digestive tract symptoms (2), headache (7), weakness (7), loss of smell (7), productive cough (7), chest pain (7)	No attack during the infection	Hypertension, favipiravir	danazol as needed
17. 45/F	PCR test	Tiredness (10), muscle pain (4), headache (6), weakness (4), loss of smell (14), dysgeusia (21), runny nose (7)	1 Extremity	None, did not require treatment	rC1-INH as needed, icatibant
18. 34/M	Close contact	Fever (3), dry cough (3), tiredness (3), muscle pain (3), gastrointestinal symptoms (1), weakness (4), loss of smell and dysgeusia (14), chest pain (7)	No attack during the infection	None, did not require treatment	pdC1-INH as needed, icatibant

*F* female, *M* male, *HAE* hereditary angioedema, *AG* antigen, *PCR* polymerase chain reaction, *pdC1-INH* plasma derived C1-INH, *rC1-INH* recombinant C1-INH, *LTP* long-term prophylaxis.

*The symptom persisted as a post covid symptom for months.

Out of 18 infected patients, 13 (72%) did not have HAE attacks during the SARS-CoV-2 infection, while 5 patients (28%) did: 3 abdominal, 3 localized on the extremities, one of 5 patients had an attack on both sites.

In the acute phase of the infection, the evaluation of the ratio (5/18) of the C1-INH-HAE patients with attacks during the infection was influenced by the facts that 4/18 (22%) patients have never had HAE attack before; 2/18 (11%) patients did not have an attack for 2 years; 1–1/18 (6%) patients did not have an attack for 8 and 15 years. If we only view the clinically active C1-INH-HAE patients, in 5/10 (50%) cases, HAE attacks occurred during the acute infection. 1/5 patients used danazol for LTP out of those who also experienced HAE attacks during the infection; and altogether 3/18 patients used androgens for LTP. This may also influence the received results.

### Survey of the post covid symptoms

8/18 patients who were infected have experienced PCS after the acute phase of the infection. The PCS occurring most often included loss of smell (3 patients), dysgeusia (2 patients), recurrent headache (1 patient), recurrent chest discomfort (1 patient), tachycardia (1 patient), hair loss (1 patient), or varicose veins in the lower extremities (1 patient). All patients with PCS, as well as 3 patients without PCS received LTP. Danazol was administered as LTP in all cases.

### Evaluation of vaccination status

From the 93 examined C1-INH-HAE patients, 73 received two vaccines (in case of Janssen Vaccine, only one), 20 patients did not receive any vaccine during the observation period. A total number of 142 doses of vaccines were administered to the patients (vaccinated min–max age: 18–79 years, median age: 49 years; unvaccinated min–max age: 18–67 years, median age: 44 years). Altogether 38 patients got Comirnaty, 11 Sinopharm, 8 Moderna COVID-19 Vaccine, 7 Gam-COVID-Vac, 5 Vaxzevria and 4 Janssen Vaccine. In addition, 4 patients received STP, 17 patients received LTP, and 2 patients received STP and LTP before the vaccination. The medication used for STP was iv. pd C1-INH in all cases. For LTP, danazol was administered in most cases, except from one patient, who used iv. pd C1-INH as LTP. In the 48 h following the vaccination, the most frequent side effects were: muscle pain (36 patients, 49%); weakness (30; 41%); malaise (26; 35%); joint pain (19; 26%), shivering (18; 25%), fever or hyperthermia (17; 23%), headache (16; 22%), local reaction (14; 20%), gastrointestinal symptoms (7; 10%), neurological symptoms (4; 5%), and respiratory symptoms (1; 1%). The symptoms experienced after the vaccine are detailed in Table 2. From the 73 vaccinated patients, 4 reported angioedematous attack after the vaccine (all 4 patients had abdominal attacks, 5%); 2 received mRNA vaccine, while 2 received vector vaccines. From these 4 patients, 1 received STP and LTP, 1 patient received only LTP, while the remaining 2 patients were not administered any prophylactic therapy. The attacks eased after the administration of iv. pd C1-INH.

**Table 2:** The vaccination against SARS-CoV-2 and the distribution of the side effects of the vaccines in hereditary angioedematous patients.

	Comirnaty	Sinopharm	Moderna-COVID-19 vaccine	Gam-COVID-Vac	Vaxzevria	Janssen Vaccine	∑
Number of vaccinated patients	38	11	8	7	5	4	73
Muscle pain	22	3	2	5	4	0	36
Weakness	15	0	4	4	4	3	30
Bad mood	13	0	4	5	3	1	26
Joint pain	11	0	2	3	3	0	19
Headache	12	0	2	1	1	0	16
Local reaction	8	1	2	2	0	1	14
GI symptoms	5	0	1	1	0	0	7
Airway symptoms	0	0	1	0	0	0	1
Neurological symptoms	3	0	1	0	0	0	4
Chest pain	1	0	0	0	0	0	1
Rash throughout the body	1	0	0	0	0	0	1
Hypertension	1	0	0	0	0	0	1
Amenorrhea	1	0	0	0	0	0	1
Tachycardia	1	0	0	0	0	0	1
Shivering	5	0	5	3	4	1	18
Hyperthermia or fever	5	0	4	4	3	1	17
HAE attack	1	0	1	1	0	1	4

The side effects occurred as follows: muscle pain (49%), weakness (41%), bad mood (36%), malaise (35%); joint pain (26%), shivering (25%), fever or hyperthermia (23%), headache (22%), local reaction (20%), gastrointestinal symptoms (10%), HAE attack (5%), neurological symptoms (5%), airway symptoms (1%), chest pain (1%), rash throughout the body (1%), hypertension (1%), amenorrhea (1%), tachycardia (1%).

*GI* gastrointestinal, *HAE* hereditary angioedema.

### The availability of C1-INH-HAE patients’ medications and the treating physician

According to 94% of the C1-INH-HAE patients who used HAE therapy (64/68 patients), the availability of the HAE medication did not change in the analyzed period; 3% stated that the availability improved (2 patients) and 3% said that the availability worsened (2 patients). The availability of HAE treating physicians during this time, according to 96% of 93 patients (89 patients), did not change; 3% reported improvement (3 patients) and 1% said it worsened (1 patient).

### Evaluation of the AE-QoL total score

The AE-QoL questionnaire was completed by 63 patients, both prior to and during the pandemic. The median of the recorded AE-QoL total score (25% and 75% percentiles) from the time prior to the pandemic (March 2019–March, 2020) is 23.53 (2.94; 37.13). The same value during the pandemic time period (September, 2020–April, 2021) was 13.23 (1.47; 33.82). Although the improving tendency of the QoL was observed during the pandemic, there was no significant difference between the two periods regarding total score (p = 0.59). In 19 cases the total score measured during the pandemic decreased with more than 6 points (the QoL improved) and in 9 cases it increased with 6 points (the QoL decreased) when compared to the survey prior to the pandemic.

## Discussion

The severity of the course of the SARS-CoV-2 infection is determined with the therapy required for the successful treatment of the infection (like need for hospitalization, need for intensive care). In our study, 5% of the infected patients required hospitalization, 5% went to the emergency care unit with their symptoms, no patient needed intensive care. In the study of Grumach et al., similar results were reported; one (7%) C1-INH-HAE patient needed hospitalization, while Bostan et al. also reported that one (11%) of the infected C1-INH-HAE patients required hospitalization, but intensive care was not required in any case.^29,30^ Although Bostan et al.^29^ did not report data regarding the course of disease, in the study of Grumach et al.^30^, the most frequent COVID-19 symptoms were anosmia (77%) and dysgeusia (77%), while these symptoms in our study were observed in 56% and 39% of the SARS-CoV-2 infected patients, respectively. In the survey of Olivares et al. 14% of all HAE patients required hospitalization during SARS-CoV-2 infection, one of them (1.8%), a 71 year-old patient with no comorbidity, lost their life during the hospitalization^31^. Veronez et al. who analyzed the data of lots of SARS-CoV-2 infected C1-INH-HAE patients (n = 69), found that 14.5% of the patients presented to the emergency care unit with their symptoms, while only 2.9% required substantial hospital care^32^.

Based on the above-mentioned data, SARS-CoV-2 infection in C1-INH-HAE patients did not require intensive therapy in the vast majority of cases. It is important to add that in the above-mentioned surveys, and in ours as well, the median age of the patients was under 50 years, and SARS-CoV-2 infection typically causes a more serious clinical picture in the older generation.

In our study, 27% of the patients experienced at least one angioedematous attack during the acute phase of the SARS-CoV-2 infection. Regarding localization, 16% of the infected patients have experienced abdominal, 22% reported an edematous attack on the extremities. In the study of Grumach et al., 50% of C1-INH-HAE patients had an edematous attack in the acute phase of the infection; 37.5% had limb edema, 12.5% had abdominal localization, while Olivares et al. measured this ratio at 45.5%, also in C1-INH-HAE patients^30,31^. In the acute phase of SARS-CoV-2 infection the ratio of patients experiencing edematous attacks in the study of Belbezier et al. was 27%; the location of the edematous attacks was abdominal in 18% and localized to the extremities in 9%. In the study of Gökmen et al., the above-mentioned ratio was 63%, they did not disclose data about the localization of the attacks, while Bostan et al. reported this ratio at 44%, 22% on the extremities, and 22% abdominal^29,33,34^.

Based on the previously mentioned results edematous attacks localized to the abdomen and the extremities were the most frequent, just like in our results. However, the ratio of the patients experiencing attacks after having been undergone the infection was higher in some studies. In case of the survey of Bostan et al., the frequency of the edematous attacks during the infection could be explained with the fact that their examined population had a higher ratio of patients who had edematous attacks in the examined year besides the acute phase of the infection, while this is not true to 44% of our population who have undergone the infection^29,30^. Gökmen et al. mention in their study that 54% of the infected patients did not use any kind of LTP despite having a higher attack frequency anyway, which may explain the outstanding results of their study^34^. In the survey of Olivares et al. 50% of all HAE patients who had an edematous attack during being infected did not use any kind of LTP, which may explain the different results in this case^31^.

In our survey, 44% of patients have experienced PCS after the SARS-CoV-2 infection, most often loss of smell/dysgeusia (16%). Fernández-de-las-Peñas et al. have summarized 34 articles mentioning post covid in their review, where they combined the populations. In this combined population 45.9% of the patients experienced PCS persisting for more than 3 months, out of which tiredness (35.3%) and shortness of breath (26.3%) were the most common. Since our results are identical with that of the review’s, there was no significant deviation in our C1-INH-HAE population in the PCS persisting for more than 3 months, when compared to the healthy population^35^.

In our survey, 5% of C1-INH-HAE patients reported an edematous attack after getting a COVID-19 vaccine; they were located in the abdomen in all cases. In contrast, in their study, Fijen et al. have reported that 16% of their patients had an attack after being vaccinated; 50% in the extremities, 40% in the abdomen. Their study, however, does not unequivocally discuss how exactly are C1-INH-HAE and nC1-INH-HAE patients divided in their population, and 6/10 of the patients who had an HAE attack after the vaccine had a poor AECT (Angioedema Control Test) result, which may convey more frequent HAE attacks or that the attacks are more difficult to treat with the applied therapy^36,37^.

Bostan et al. received similar results to ours, regarding the survey on the QoL, although they only evaluated the QoL of 9 C1-INH-HAE and 1 nC1-INH-HAE patients who have undergone SARS-CoV-2 infection. Three tests from different times were compared, the first prior to the infection, the second during the acute phase of the infection, and the third 3 months after the acute phase. No significant difference was found between the data of the three questionnaires^29^. In contrast, in our study, we found a slightly improving QoL with the decrease in the median of the total score during the pandemic. There may be multiple reasons why we found slightly improving QoL related to angioedematous symptoms compared to the period prior to the pandemic. One of them may be that the AE-QoL questionnaire can evaluate the 4 weeks before the completion with the most certainty. As the recall period increases, so does the bias of the test^27^. The other reason of the improving QoL may be that in our study, we analyzed the second and third wave of COVID-19 in Hungary, which had very similar lockdowns and restrictions as the first wave. It may be possible that with time, people’s approach both to SARS-CoV-2 and the restrictions introduced due to the pandemic changed, and the resulting situation caused less fear and uncertainty in them.

## Conclusions

Our study investigated a significantly high number of HAE patients, analyzing not only the course of COVID-19, but also if vaccinations against SARS-CoV-2 pose a specific risk to HAE patients, including vector based, live attenuated, as well as mRNA vaccines. To our knowledge, this is also the first study that investigated COVID-19 in HAE patients from the Hungarian population.

Based on our research, we assume that SARS-CoV-2, similarly to other infections, may induce HAE attacks in certain cases, but their course is mostly similarly severe to the attacks the patient is used to, and they can be treated successfully as well. Although the median age (44.5) of the analyzed HAE population that underwent SARS-CoV-2 infection was not in the endangered age group, the number of infected patients in the population is small^18^, and only 4 patients have a comorbidity of internal medicine, so based on the received results, it seems that HAE does not aggravate the acute infection of SARS-CoV-2 infection. Furthermore, despite the limitations of our survey, we propose that since HAE attacks occurring during the acute phase of the infection always had a milder course, the two diseases do not aggravate each other’s course, despite the overlaps in their pathomechanisms.

During our survey, out of 73 patients, HAE attacks occurred as the acute side effect of the SARS-CoV-2 vaccines in only 4 patients, in the days after the administration of the vaccine. We established that neither mRNA, vector based nor live attenuated SARS-CoV-2 vaccines pose a higher risk to C1-INH-HAE patients, than to the average population.

According to our patients, the availability of HAE physicians and HAE medications did not change in the analyzed pandemic period.

Since there was no significant difference between the attack number prior to and during the pandemic, and we did not find any significant difference in the QoL in between the two periods, the findings of this study indicate that the second and third wave of the Hungarian COVID-19 epidemic did not significantly influence the clinical picture of C1-INH-HAE.

## Data Availability

The datasets generated and/or analysed during the current study are not publicly available due to individual privacy but are available from the corresponding author on reasonable request.

## Abbreviations

ACE: Angiotensin-converting enzyme
AECT: Angioedema control test
AE-QoL questionnaire: Angioedema quality of life questionnaire
ARDS: Acute respiratory distress syndrome
Bk: Bradykinin
B2-R: Bradykinin 2 receptor
C1-INH: C1-inhibitor
C1-INH-HAE: Hereditary angioedema due to C1 inhibitor deficiency
COVID-19: Coronavirus disease 2019
HAE: Hereditary angioedema
LTP: Long-term prophylaxis
MCID: Minimal clinically important difference
mRNA: Messenger ribonucleic acid
nC1-INH-HAE: Hereditary angioedema with normal C1 inhibitor
NET: Neutrophil extracellular traps
PCR: Polymerase chain reaction
PCS: Post COVID symptoms
QoL: Quality of life
RNA: Ribonucleic acid
SARS-CoV-2: Severe acute respiratory syndrome coronavirus 2
STP: Short-term prophylaxis

## References

[1] Hahn J, Hoffmann TK, Bock B, Nordmann-Kleiner M, Trainotti S, Greve J (2017). Angioedema. Deutsches Ärzteblatt Int..

[2] Kaplan AP, Greaves MW (2005). Angioedema. J. Am. Acad. Dermatol..

[3] Cicardi M, Aberer W, Banerji A, Bas M, Bernstein JA, Bork K (2014). Classification, diagnosis, and approach to treatment for angioedema: consensus report from the Hereditary Angioedema International Working Group. Allergy.

[4] Germenis AE, Speletas M (2016). Genetics of hereditary angioedema revisited. Clin. Rev. Allergy Immunol..

[5] Proper SP, Lavery WJ, Bernstein JA (2020). Definition and classification of hereditary angioedema. Allergy Asthma Proc..

[6] Longhurst HJ, Bork K (2019). Hereditary angioedema: An update on causes, manifestations and treatment. Br. J. Hosp. Med. (Lond)..

[7] Kőhalmi KV, Veszeli N, Cervenak L, Varga L, Farkas H (2017). A novel prophylaxis with C1-inhibitor concentrate in hereditary angioedema during erythema marginatum. Immunol. Lett..

[8] Alonso MLO, Valle SOR, Tórtora RP, Grumach AS, França AT, Ribeiro MG (2020). Hereditary angioedema: A prospective study of a Brazilian single-center cohort. Int. J. Dermatol..

[9] Zotter Z, Csuka D, Szabó E, Czaller I, Nébenführer Z, Temesszentandrási G (2014). The influence of trigger factors on hereditary angioedema due to C1-inhibitor deficiency. Orphanet. J. Rare Dis..

[10] Kaplan AP, Pawaskar D, Chiao J (2020). C1 inhibitor activity and angioedema attacks in patients with hereditary angioedema. J. Allergy Clin. Immunol. Pract..

[11] Busse PJ, Christiansen SC, Riedl MA (2021). US HAEA medical advisory board 2020 guidelines for the management of hereditary angioedema. J. Allergy Clin. Immunol. Pract..

[12] Fijen LM, Bork K, Cohn DM (2021). Current and prospective targets of pharmacologic treatment of hereditary angioedema types 1 and 2. Clin. Rev. Allergy Immunol..

[13] Alsharif W, Qurashi A (2021). Effectiveness of COVID-19 diagnosis and management tools: A review. Radiography (Lond)..

[14] Kirtipal N, Bharadwaj S, Kang SG (2020). From SARS to SARS-CoV-2, insights on structure, pathogenicity and immunity aspects of pandemic human coronaviruses. Infect. Genet. Evol..

[15] Hu B, Huang S, Yin L (2021). The cytokine storm and COVID-19. J. Med. Virol..

[16] Umakanthan S, Sahu P, Ranade AV, Bukelo MM, Rao JS, Abrahao-Machado LF (2020). Origin, transmission, diagnosis and management of coronavirus disease 2019 (COVID-19). Postgrad. Med. J..

[17] Merkely B, Szabó AJ, Kosztin A, Berényi E, Sebestyén A, Lengyel C (2020). Novel coronavirus epidemic in the Hungarian population, a cross-sectional nationwide survey to support the exit policy in Hungary. Geroscience..

[18] Soriano JB, Murthy S, Marshall JC, Relan P, Diaz JV (2022). A clinical case definition of post-COVID-19 condition by a Delphi consensus. Lancet Infect. Dis..

[19] Davido B, Seang S, Tubiana R, de Truchis P (2020). Post-COVID-19 chronic symptoms: A postinfectious entity?. Clin. Microbiol. Infect..

[20] Sher L (2021). Post-COVID syndrome and suicide risk. QJM..

[21] Meo SA, Bukhari IA, Akram J, Meo AS, Klonoff DC (2021). COVID-19 vaccines: comparison of biological, pharmacological characteristics and adverse effects of Pfizer/BioNTech and Moderna Vaccines. Eur. Rev. Med. Pharmacol. Sci..

[22] Hernández AF, Calina D, Poulas K, Docea AO, Tsatsakis AM (2021). Safety of COVID-19 vaccines administered in the EU: Should we be concerned?. Toxicol. Rep..

[23] Xu Y, Liu S, Zhang Y, Zhi Y (2020). Does hereditary angioedema make COVID-19 worse?. World Allergy Organ. J..

[24] Garvin MR, Alvarez C, Miller JI, Prates ET, Walker AM, Amos BK (2020). A mechanistic model and therapeutic interventions for covid-19 involving a ras-mediated bradykinin storm. Elife..

[25] van Mulukom V, Muzzulini B, Rutjens BT, van Lissa CJ, Farias M (2021). The psychological impact of threat and lockdowns during the COVID-19 pandemic: Exacerbating factors and mitigating actions. Transl. Behav. Med..

[26] Dávid B, Szabó T, Huszti É, Bukovics I (2021). A COVID–19 járvány hatása a leghátrányosabb helyzetű településeken élők mindennapjaira: ahogy a hátrányos helyzetűek és a szociális szolgáltatásokat nyújtók látják. Scientia et Securitas..

[27] Weller K, Groffik A, Magerl M, Tohme N, Martus P, Krause K (2012). Development and construct validation of the angioedema quality of life questionnaire. Allergy..

[28] Weller K, Magerl M, Peveling-Oberhag A, Martus P, Staubach P, Maurer M (2016). The Angioedema Quality of Life Questionnaire (AE-QoL) - assessment of sensitivity to change and minimal clinically important difference. Allergy..

[29] Can Bostan O, Tuncay G, Damadoglu E, Karakaya G, Kalyoncu AF (2021). Effect of COVID-19 on hereditary angioedema activity and quality of life. Allergy Asthma Proc..

[30] Grumach AS, Goudouris E, Dortas Junior S, Marcelino FC, Alonso MLO, Martins RO (2021). COVID-19 affecting hereditary angioedema patients with and without C1 inhibitor deficiency. J. Allergy Clin. Immunol. Pract..

[31] Olivares MM, Zwiener RD, Panqueva LML, Contreras Verduzco FA, Mansour E, Rodriguez JA (2022). COVID-19 triggers attacks in HAE patients without worsening disease outcome. J. Allergy Clin. Immunol. Pract..

[32] Veronez CL, Christiansen SC, Smith TD, Riedl MA, Zuraw BL (2021). COVID-19 and hereditary angioedema: Incidence, outcomes, and mechanistic implications. Allergy Asthma Proc..

[33] Belbézier A, Arnaud M, Boccon-Gibod I, Pelletier F, McAvoy C, Gobert D (2021). COVID-19 as a trigger of acute attacks in people with hereditary angioedema. Clin. Exp. Allergy..

[34] Mete Gökmen N, Kuman Tunçel O, Boğatekin G, Bulut G, Demir S, Gelincik A (2021). Psychiatric and clinical characteristics of hereditary angioedema patients who experienced attacks during COVID-19. J. Investig. Allergol. Clin. Immunol..

[35] Fernández-de-Las-Peñas C, Palacios-Ceña D, Gómez-Mayordomo V, Florencio LL, Cuadrado ML, Plaza-Manzano G (2021). Prevalence of post-COVID-19 symptoms in hospitalized and non-hospitalized COVID-19 survivors: A systematic review and meta-analysis. Eur. J. Intern. Med..

[36] Fijen LM, Levi M, Cohn DM (2021). COVID-19 vaccination and the risk of swellings in patients with hereditary angioedema. J. Allergy Clin. Immunol. Pract..

[37] Weller K, Donoso T, Magerl M, Aygören-Pürsün E, Staubach P, Martinez-Saguer I (2020). Validation of the angioedema control test (AECT)-A patient-reported outcome instrument for assessing angioedema control. J. Allergy Clin. Immunol. Pract..

